# Effects of Astragalus Polysaccharides on CD8+ Tissue-Resident Memory T Cells in Mice with Herpes Simplex

**DOI:** 10.1155/2022/7729136

**Published:** 2022-03-28

**Authors:** Liqing Shi, Cong Zhang, Lihao Liu, Zhaoqing Xi, Min Chen

**Affiliations:** ^1^Department of Dermatology, Hospital for Skin Diseases (Institute of Dermatology), Chinese Academy of Medical Sciences & Peking Union Medical College, Nanjing, Jiangsu 210042, China; ^2^Affiliated Hospital of Nanjing University of Chinese Medicine, Jiangsu Province Hospital of Chinese Medicine, Nanjing 210029, China; ^3^Nanjing University of Chinese Medicine, Nanjing 210023, China

## Abstract

**Objective:**

This study aimed to explore whether astragalus polysaccharides (APS) could treat herpes simplex by increasing tissue-resident memory CD8+ T cells (CD8+ TRM cells) and analyze its potential mechanism using the network pharmacologic approach.

**Methods:**

C57BL/6J male mice aged 6–8 weeks were divided into a model group with HSV-1 infection treated by saline, a control group without HSV-1 infection but treated by saline, and an APS group with HSV-1 infection treated by APS. Clinical signs were observed, and the disease score was recorded every day. The skin lesions on day 9 after infection were taken for flow cytometric analysis to evaluate CD8+ TRM cells. Network pharmacologic analysis was performed to select the potential protein targets of astragalus associated with herpes simplex. Besides, Gene Ontology (GO) analysis and Kyoto Encyclopedia of Genes and Genomes (KEGG) pathway analysis were performed. The peripheral blood from the retroorbital venous plexus was collected to evaluate the levels of serum interferon-*γ* (IFN-*γ*) and interleukin 12 (IL-12). The comparisons of clinical signs, the disease score, CD8+ TRM cells, the serum IFN-*γ*, and IL-12 levels were performed among the three groups.

**Results:**

Compared with the model group, the disease score in the APS group was significantly lower (*p* < 0.05). On the day 9 after HSV-1 infection, there was no significant difference in the body weight of mice among the three groups. However, compared with the control group or model group, the spleen weight in the APS group increased significantly (*p* < 0.05). The surface antigens of CD8+ TRM cells had no significant difference between the control group and the model group, while compared with the model group, the surface antigens of CD8 (*p* < 0.05), CD69 (*p* < 0.05), and CD103 (*p* < 0.05) in the APS group increased significantly. Moreover, the serum IL-12 (*p* < 0.05) and IFN-*γ* (*p* < 0.01) levels in the APS group increased significantly compared with the model group.

**Conclusion:**

Our study suggested that APS could alleviate the symptoms of the mice infected with HSV-1, and CD8+ TRM cells in the skin lesions and the levels of IL-12 and IFN-*γ* in the serum of mice with HSV-1 infection increased after the APS treatment, of which the specific underlying mechanism requires further experiments to clarify. In addition, the antiviral effect of APS might be worthy of further development and utilization.

## 1. Introduction

Based on memory T cell migration patterns, memory T cells are classified into recirculating memory T-cell population such as effector memory (TEM) and central memory (TCM) cells, and the nonrecirculating T-cell population such as tissue-resident memory (TRM) cells that are retained in peripheral tissues without recirculating [1]. Although recirculating memory T cells provide enhanced protection against pathogen attack, they usually fail to provide adequate protection when infection is localized to the peripheral tissues, and yet, TRM cells in peripheral tissues can provide superior immune protection [2]. It has been reported that, for herpes simplex virus (HSV) infection, local inflammation in the skin and mucosa can recruit effector immune cells and make them transform into the TRM cells, then, the CD8+ TRM cells stay in these barrier tissues and provide a long-term protection against local HSV infection [3–6].

HSV-1 is a neurotropic herpes virus which can lead to a life-long infection in a human host. Following primary infection, this virus can establish a latent infection in sensory neuronal cells [7]. Once the host has low immunity, HSV-1 can reactivate to cause more serious herpes simplex [8]. It has been reported that CD8+ T cells with the characteristics of TRM cells take a crucial part in inhibiting HSV-1 reactivation. During the recurrence of herpes simplex, CD8+ TRM cells can accumulate at the dermal-epidermal junction to prevent the release of HSV from neuronal endings to the mucosa [3]. Unfortunately, so far, it is still difficult for the ex vivo expansion of TRM cells. The search for potential drugs that can induce TRM cell proliferation in vivo has become critical.

**Figure 1:**
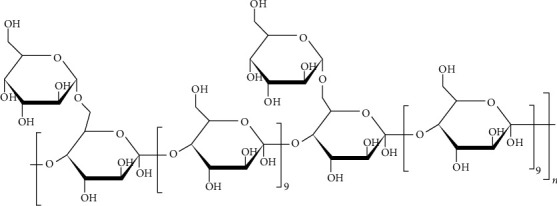
Characteristic structure of APS.

Previous studies have suggested that medicinal herbs and plants have strong potential values for improving human health and preventing and treating diseases, such as inhibiting tumor growth [9, 10], relieving central and peripheral pain [11], antidepression [12], and preventing and treating neurological disorders [13], gastrointestinal diseases [14], viral infections, and even COVID-19 [15]. *Astragalus membranaceus* Bunge is a commonly used Chinese medicinal herb and also used as food material with a history of more than 2000 years. Astragalus polysaccharide (APS) is the main active ingredient extracted from *Astragalus membranaceus* Bunge, which is characterized by a 1,4-linked dextran backbone with a 1,6-linked branch in every 10 residues (Figure 1) [16]. APS is composed of a series of monosaccharides, including rhamnose, galactose, glucose, and arabinose and has a variety of pharmacological and physiological functions, including immunomodulatory, anticancer, and hepatoprotective effects [17, 18]. However, whether APS has antiviral effects is still unclear. Therefore, our study aimed to explore whether APS could treat herpes simplex by increasing CD8+ TRM cells and its potential mechanism using network pharmacologic analysis.

**Figure 2:**
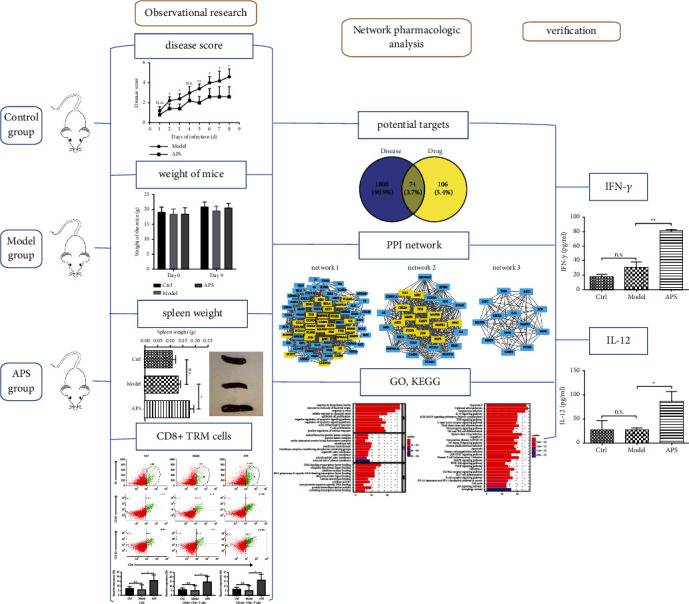
Flow chart of this research.

The flow chart of this study is as follows (Figure 2).

## 2. Materials and Methods

### 2.1. Reagents and Materials

Model 5417R low-temperature centrifuge (Eppendorf, Germany), HH-6 constant temperature water bath (Shanghai Jinghong Experimental Equipment Co., Ltd.), Sony ILCE-6300 digital camera (Sony, Japan), Revco ULT ultralow temperature refrigerator (Thermo Fisher, USA), and Flow sorter (Beckman Coulter FC500) were used in this study.

APS with the batch number of C11M7Y10255 (Shanghai Yuanye Biological Technology Co., Ltd.) was used to prepare the 25 mg/ml solution by adding normal saline. Every mouse was administrated 250 mg/kg by intraperitoneal injection. The HSV-1 McKare strain was obtained from Director Hu Kai, Drum Tower Hospital, Nanjing University School of Medicine. Main reagents were as follows: normal saline, Hanks, red blood cell lysate, BSA powder, collagenase A, DNase I, and flow antibody (CD8a FITC 53-6.7, CD103 PE 2E7, and CD69 PerCP-Cy5.5 H1.2F3).

### 2.2. Grouping and Modeling

The C57BL/6J male mice aged 6–8 weeks were divided into following groups: Group 1 (model group) with HSV-1 infection treated by saline, Group 2 (control group) receiving the same treatment with Group 1 but without HSV-1 infection, and Group 3 (APS group) with HSV-1 infection treated by APS (Table 1).

The specific operations are as follows.

The mice were anesthetized by intraperitoneal injection and then, the hair on right back of mice was removed. Next, we nicked the skin 20 times using a 27-gauge needle to incubate 50 *µ*L of HSV-1 (containing 7^*∗*^10^6^ PFU/ml) was incubated into the skin of an area of about 1 cm^2^ in each mouse. During the following days, each mouse was observed for clinical signs and was scored based on the following standards every day:

  0 point: no signs of infection;
  1 point: local and barely noticeable vesicles;
  2 points: scattered small blisters;
  3 points: large areas of small blisters;
  4 points: small vesicles zoster;
  5 points: large areas of ulcer;
  6 points: large areas of herpes zoster-like ulcers;
  7 points: hind limb paralysis or death.

### 2.3. Detection of the Proportion of CD8+ TRM Cells by Flow Cytometry

HSV-1 was inoculated into the skin of mice, and that day was recorded as the day 0 after infection. The skin lesions were taken for flow cytometric analysis on day 9 after infection to calculate the proportion of CD8+ TRM cells by detecting their surface phenotypes (CD8, CD69, and CD103). The skin lesions were cut into small pieces and incubated in Hanks Balanced Salt Solution (HBSS) containing 1 mg/mL collagenase A and 40 *μ*g/mL DNase I at 37°C for 30 min. Then, we centrifuged the cell suspension using a 70 *μ*m nylon cell strainer (500 g; 10 min; 4°C) to collect the cells, washed the cells with phosphate buffered saline (PBS), and stained the cells with flow antibody for 30 min. Furthermore, the cells were resuspended in 100 *μ*L/tube of PBS staining buffer containing 1% BSA, and incubated with an antibody concentration of 1 *μ*g/100 *μ*L at 4°C for 15 min. After staining, centrifuging (500 g; 10 min; 4°C) and discarding the supernatant, we washed the cells with 1 mL 1% BSA-PBS, centrifuged them (350 g; 4°C; 5 min), and discarded the supernatant, again. Finally, the cells were resuspended in 300 *μ*L of 1% BSA-PBS, and tested.

### 2.4. Determination of Serum Interferon-*γ* (IFN-*γ*) and Interleukin 12 (IL-12) Concentrations in Mice

The peripheral blood from the retroorbital venous plexus in the mice was collected and left to stand for 2 hours. The blood was centrifuged at 3000 rpm for 10 min at room temperature to obtain serum, and then, the serum was stored in aliquots in an ultralow temperature refrigerator at −80°C for later use. We used the interferon-*γ* (IFN-*γ*) and interleukin-12 (IL-12) enzyme-linked immunoassay kit purchased from LinkTech Co., Ltd. for detection. The amount of serum per well of the ELISA plate was 20 *μ*L. The experimental operation was carried out in strict accordance with the instructions. After adding the color reagent, we read the OD value at the specified wavelength using a microplate reader, and then calculated the content of each cytokine according to the standard curve and the formula in the manual.

### 2.5. Network Pharmacologic Analysis

#### 2.5.1. Identification of Targets for Astragalus and Herpes Simplex

All components of astragalus were downloaded from the Traditional Chinese Medicines for Systems Pharmacology database (TCMSP, https://tcmspw.com/tcmsp.php) [19]. Based on the screening criteria of oral bioavailability (OB) ≥30%, drug-likeness (DL) ≥0.18 [20], we selected the active components of astragalus. The protein targets of the active components of astragalus were retrieved from the TCMSP database. The herpes simplex disease-related targets were collected from the GeneCards database [21] (https://www.genecards.org), Online Mendelian Inheritance in Man (OMIM) (https://omim.org/), PharmGkb (https://www.pharmgkb.org/), and Therapeutic Target Database (TTD) (http://db.idrblab.net/ttd/). We used Drug Bank [22] (http://www.drugbank.ca), Universal Protein (UniProt) [23] (https://www.uniprot.org), and PubChem [24] (http://pubchem.ncbi.nlm.nih.gov) to match the drug targets and disease targets. The intersection set of the protein targets of the active components of astragalus and the herpes simplex disease-related targets was visualized by a Venn diagram.

#### 2.5.2. Function and Pathway Enrichment Analyses

Gene Ontology (GO) functional enrichment analysis and Kyoto Encyclopedia of Genes and Genomes (KEGG) pathway enrichment analysis were performed using the “clusterProfiler,” “enrichplot,” and “ggplot2” packages of R language. GO results included molecular function (MF), biological process (BP), and cellular component (CC). FDR <0.05 was considered statistically significant.

#### 2.5.3. Construction of Protein-Protein Interaction (PPI) Network and Identification of Hub Genes

We used STRING website to construct PPI network for the analysis of the interactions between the genes in the intersection set, and used CytoNCA and Cytoscape plug-in to identify the hub genes.

## 3. Results

### 3.1. Effects of APS on Mice Infected with HSV-1

**Figure 3:**
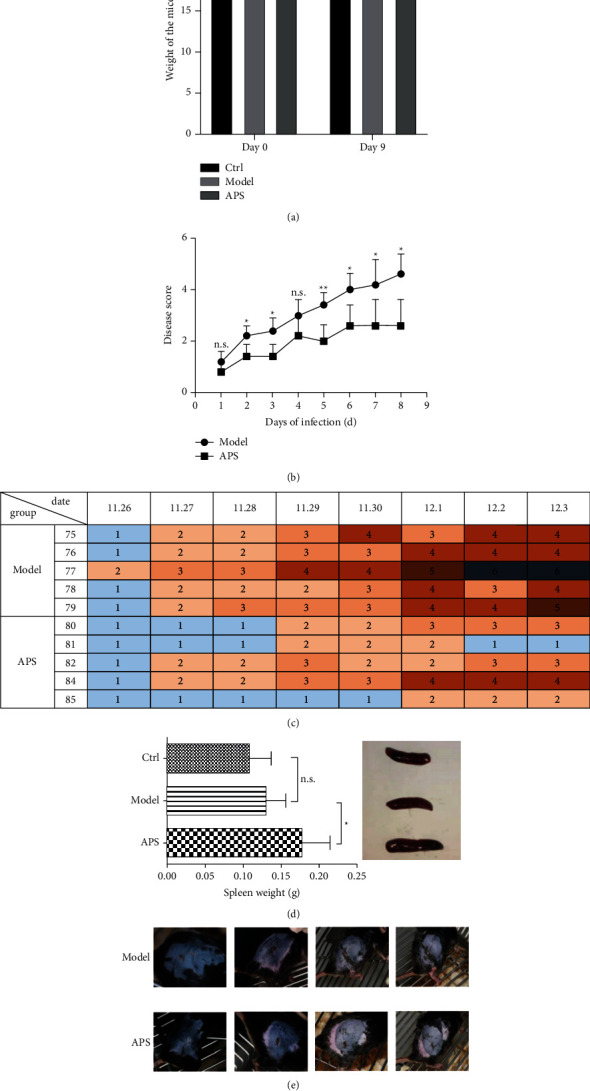
Effects of APS on mice infected with HSV-1. (a) There was no significant difference in the body weight of the mice among the three groups. (b) The disease score of the mice in the APS group was significantly lower than that in the model group (^*∗*^*p* < 0.05, ^*∗∗*^*p* < 0.01, and n.s.: no significant difference). (c) The disease scores of the mice during the eight days after infection in the APS group and model group were shown. (d) On the day 9 after infection, the spleen weight in the APS group was significantly larger than that in the other two groups. (e) Skin lesions of the mice in the APS group and model group were shown.

We found that APS could relieve the symptoms of HSV-1 infection in mice.  Figure 3(e) showed that every mouse infected with HSV-1 had obvious symptoms to varying degrees. The disease scores of the mice during the eight days after infection were shown in Figure 3(c), ranging from 1 to 6 points. Compared with the model group, the disease score in the APS group was significantly lower (Figure 3(b)). On the day 9 after HSV-1 infection, there was no significant difference in the body weight of mice among the control group, model group, and APS group (Figure 3(a)). Besides, Figure 3(d) showed that there was no significant difference in the spleen weight between the control group and the model group, while compared with the control group or model group, the spleen weight in the APS group increased significantly.

### 3.2. Effects of APS on CD8+ TRM Cells in Mice Infected with HSV-1

**Figure 4:**
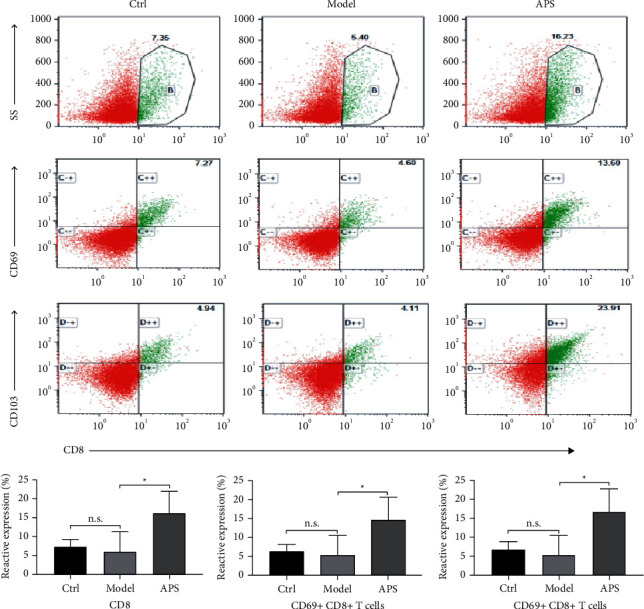
Compared with the control group or model group, the surface antigens of CD8 (*p* < 0.05), CD69 (*p* < 0.05), and CD103 (*p* < 0.05) in the APS group increased significantly (^*∗*^*p* < 0.05, n.s.: no significant difference).

The results of the flow cytometric suggested that the surface antigens of CD8+ TRM cells including CD8, CD69, and CD103, had no significant difference among the three groups, while compared with the model group, the surface antigens of CD8 (*p* < 0.05), CD69 (*p* < 0.05), and CD103 (*p* < 0.05) in the APS group increased significantly (Figure 4).

### 3.3. Identification of Main Active Components of Astragalus and the Corresponding Targets

According to the screening criteria of OB (≥30) and DL (≥0.18), 20 main active components of astragalus were obtained (Table 2). A total of 180 targets for the 20 active components were collected from TCMSP.

### 3.4. Visual Analysis of the “Astragalus-Herpes Simplex” Targets

**Figure 5:**
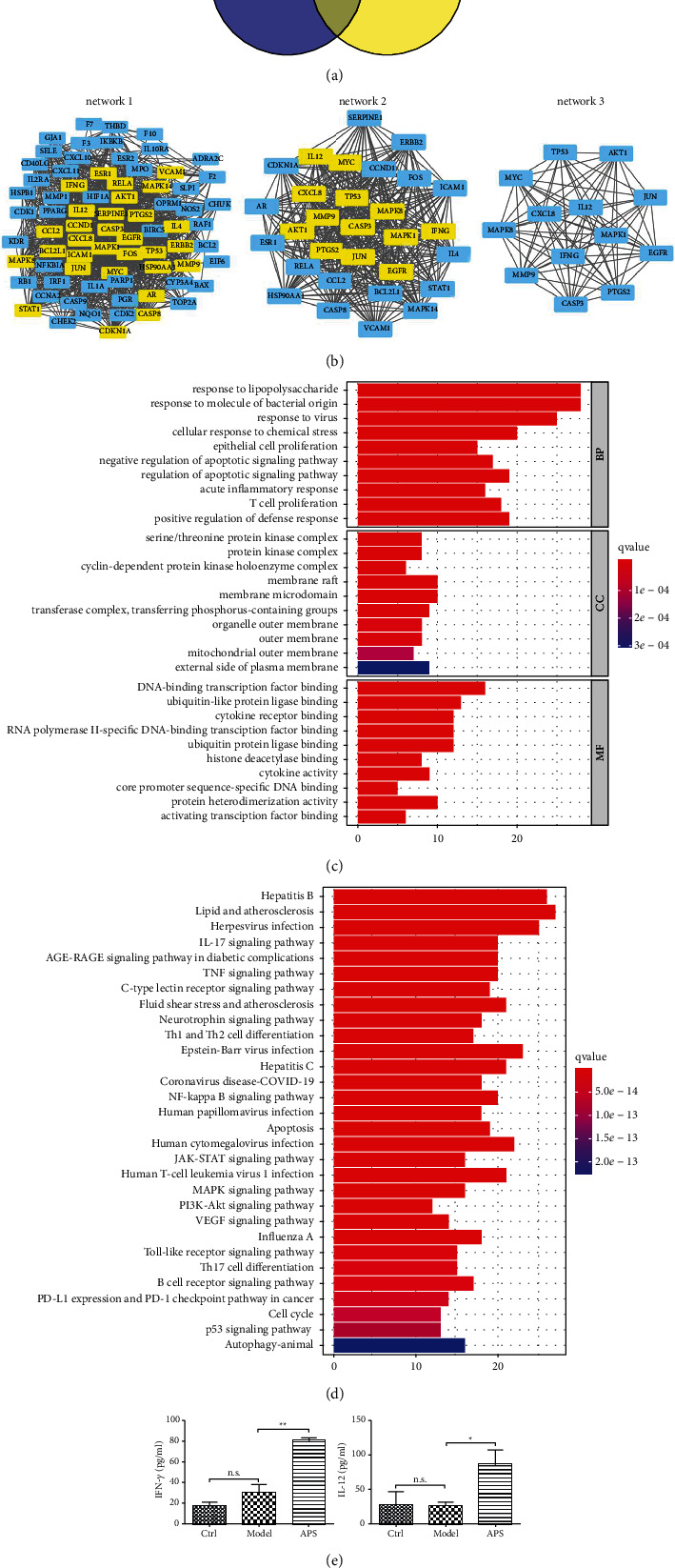
(a) The Venn diagram showed the intersection set of drug targets and disease targets. (b) The PPI network was constructed by STRING and the hub proteins were identified by the CytoNCA plugin. (c) The selected top ten of GO-BP, GO-CC, and GO-MF were visualized. (d) The selected top 30 pathways of the KEGG enrichment results were visualized. (e) The levels of IFN-*γ* and IL-12 in the APS group were significantly higher than those in the model group.

A total of 1874 potential targets for herpes simplex were obtained. The Venn diagram of drug targets and disease targets showed 74 “Astragalus-herpes simplex” targets (Figure 5(a)). These 74 targets were potential effective targets of Astragalus associated with herpes simplex. Then, they were input into the STRING database to obtain the PPI network, and the top hub proteins (CXCL8, IL-12, IFN-*γ*, MAPK1, JUN, EGFR, PTGS2, CASP3, MAPK8, MYC, TP53, AKT1, and MMP9) were identified (Figure 5(b)), which might play roles in the treatment of herpes simplex by astragalus.

### 3.5. GO Function and KEGG Pathway Enrichment Analyses

The top ten of GO-BP, GO-CC, and GO-MF were selected for visual analysis (Figure 5(c)), which revealed that these 74 genes were mainly related to the defense against bacteria and viruses, cell apoptosis, T cell proliferation, and other immune responses. KEGG enrichment analysis suggested the high enrichment of various viral infection pathways and multiple immune related pathways, such as IL-17 signaling pathway, TNF signaling pathway, NF-*κ*B signaling pathway, and JAK-STAT signaling pathway (Figure 5(d)), indicating that these pathways might be potential targets of astragalus against herpes simplex virus. Of these 74 targets, IFN-*γ* and IL-12 were enriched in various immune-related pathways.

### 3.6. Changes of Serum IL-12 and IFN-*γ* Levels in Mice

On the day 9 after infection, there was no significant difference in the serum IFN-*γ* and IL-12 levels between the model group and the control group, while compared with the model group, the serum IL-12 and IFN-*γ* levels in the APS group increased significantly (IL-12: *p* < 0.05, IFN-*γ*:*p* < 0.01) (Figure 5(e)).

## 4. Discussion

*Astragalus membranaceus* Bunge, as a traditional Chinese medicine for enhancing immunity, has been put into clinical practices for more than 2,000 years to prevent and treat exogenous pathogens in China. APS is the main active ingredient of astragalus, and has antivirus, antitumor, antiaging, antiradiation, antistress and antioxidant effects, which can enhance the activity of macrophages and natural killer (NK) cells, and promote the proliferation and differentiation of B lymphocytes and T lymphocytes [17, 18]. Our study discussed the effects of APS on herpes simplex virus infection from the perspective of immunity.

In our study, on the day 9 after infection, the size of the spleen of the mice in the APS group was significantly larger than that in the model group, suggesting that the immune function of mice in the APS group, especially T cell function, might be activated. Previous studies have showed that memory T cells can appear during the first skin infection in mice [25, 26]. However, in our present study, due to the short infection time, the increase of surface antigens of CD8+ TRM cells (CD8, CD69, and CD103) was not observed in the skin lesions of the mice in the model group. Compared with the model group, the surface antigens of CD8+ TRM cells in the APS group increased significantly (*p* < 0.05), suggesting that APS induced the production of CD8+ TRM cells in vivo.

Subsequently, network pharmacological analysis suggested that IL-12 and IFN-*γ* might be potential targets for the antiviral effect of *Astragalus membranaceus*, and the ELISA experiment showed that IL-12 and IFN-*γ* increased significantly in the serum of mice in the APS group. IL-12 is a heterodimeric proinflammatory cytokine and is the most active cytokine for activating NK cells and cytotoxic T lymphocyte [3]. The production of IFN-*γ* is an essential link between nonspecific immune response and specific immune response. It has also been reported that IL-12 and IFN-*γ* can synergistically enhance the body's immunity against viruses and tumors [27, 28]. Thus, whether there is a certain connection between the increase of IL-12 and IFN-*γ* levels and the increase of CD8+ TRM cells in the APS group still needs further experimental confirmation in the future.

CD8+ TRM cells have attracted extensive attentions in recent years, especially in the field of dermatology [29–31]. Recent studies have elucidated the mechanism of TRM cells action. In the absence of antigens, TRM cells can effectively recognize previously infected tissues to quickly clear infected cells upon reinfection. During the immune surveillance phase, the dendritic arms of TRM cells are in contact with a large number of potentially infected cells, thereby making TRM cells remain activated. After recognizing the infected cells, CD8+ TRM cells proliferate rapidly and release perforin and granzyme B to mediate the apoptosis of the infected cells. At the same time, TRM cells induce a wide range of antiviral programs, such as the release of cytokines, especially IFN-*γ*, thereby enhancing immunity, clearing HSV infection, and enhancing resistance to other pathogens [2, 3]. Although a strong antiviral effect, CD8+ TRM cells cannot be cultured in vitro, and few drugs can directly stimulate the production of TRM cells in the body. In our study, it was found that the costimulation of APS and HSV-1 in mice could significantly increase the production of local CD8+ TRM cells, inhibit pathological changes, and enhance the immunity within only 8 days, enlightening us that APS might be effective for preventing infections caused by other viruses, such as human papillomavirus (HPV), which needs to be further explored by much more experiments in the future.

In addition to its antiviral effects with fewer side effects, APS might also be used as an adjuvant for vaccines to prevent virus infection more efficiently. Its possible broad-spectrum antiviral efficacy might effectively improve the anti-infection ability of the population.

## 5. Conclusion

Our study suggested that APS could alleviate the symptoms of the mice infected with HSV-1, and CD8+ TRM cells in the skin lesions and the levels of IL-12 and IFN-*γ* in the serum of mice with HSV-1 infection increased after the APS treatment, of which the specific underlying mechanism requires further experiments to clarify. In addition, the antiviral effect of APS might be worthy of further development and utilization.

## Figures and Tables

**Table 1:** Grouping and treatment of mice.

Groups	*N*	Dose	Treatment
The control group	5	10 ml/kg	Intraperitoneal injection of saline without local infection of HSV
The model group	5	50 *μ*l	Local infection of HSV
The APS group	5	250 mg/kg (10 ml/kg)	Intraperitoneal injection of APS + local infection of HSV

**Table 2:** Main active ingredients of astragalus.

Mol ID	Molecule name	OB (%)	DL
MOL000211	Mairin	55.37707338	0.7761
MOL000239	Jaranol	50.82881677	0.29148
MOL000296	Hederagenin	36.91390583	0.75072
MOL000033	(3S,8S,9S,10R,13R,14S,17R)-10,13-dimethyl-17-[(2R,5S)-5-propan-2-yloctan-2-yl]-2,3,4,7,8,9,11,12,14,15,16,17-dodecahydro-1H-cyclopenta[a]phenanthren-3-ol	36.22847056	0.78288
MOL000354	Isorhamnetin	49.60437705	0.306
MOL000371	3,9-di-O-Methylnissolin	53.74152673	0.47573
MOL000374	5′-Hydroxyiso-muronulatol-2′,5′-di-O-glucoside	41.71766574	0.69251
MOL000378	7-O-Methylisomucronulatol	74.68613752	0.29792
MOL000379	9,10-dimethoxypterocarpan-3-D-glucoside	36.73668801	0.9243
MOL000380	(6aR,11aR)-9,10-dimethoxy-6a,11a-dihydro-6H-benzofurano[3,2-c]chromen-3-ol	64.25545452	0.42486
MOL000387	Bifendate	31.09782391	0.66553
MOL000392	Formononetin	69.67388061	0.21202
MOL000398	Isoflavanone	109.9866565	0.29572
MOL000417	Calycosin	47.75182783	0.24278
MOL000422	Kaempferol	41.88224954	0.24066
MOL000433	FA	68.96043622	0.7057
MOL000438	(3R)-3-(2-hydroxy-3,4-dimethoxyphenyl) chroman-7-ol	67.66747949	0.26479
MOL000439	Isomucronulatol-7,2′-di-O-glucosiole	49.28105539	0.62065
MOL000442	1,7-Dihydroxy-3,9-dimethoxy pterocarpene	39.04541112	0.47943
MOL000098	Quercetin	46.43334812	0.27525

## Data Availability

All data generated or analyzed during this study are included in this article. The raw data supporting the conclusions of this manuscript will be made available by the authors, without undue reservation, to any qualified researcher.
